# Body and the Senses in Spatial Experience: The Implications of Kinesthetic and Synesthetic Perceptions for Design Thinking

**DOI:** 10.3389/fpsyg.2022.864009

**Published:** 2022-04-07

**Authors:** Jain Kwon, Alyssa Iedema

**Affiliations:** Interior Architecture and Design, Department of Design and Merchandising, Colorado State University, Fort Collins, CO, United States

**Keywords:** design thinking model, spatial perception, interior design and architecture, kinesthetic perception, synesthetic perception, sensory experience, multisensory design, embodied design

## Abstract

Human perception has long been a critical subject of design thinking. While various studies have stressed the link between thinking and acting, particularly in spatial experience, the term “design thinking” seems to disconnect conceptual thinking from physical expression or process. Spatial perception is multimodal and fundamentally bound to the body that is not a mere receptor of sensory stimuli but an active agent engaged with the perceivable environment. The body apprehends the experience in which one’s kinesthetic engagement and knowledge play an essential role. Although design disciplines have integrated the abstract, metaphoric, and visual aspects of the body and its movement into conceptual thinking, studies have pointed out that design disciplines have emphasized visuality above the other sensory domains and heavily engaged with the perception of visual configurations, relying on the Gestalt principles. Gestalt psychology must be valued for its attention to a whole. However, the theories of design elements and principles over-empathizing such visuality posit the aesthetics of design mainly as visual value and understate other sensorial and perceptual aspects. Although the visual approach may provide a practical means to represent and communicate ideas, a design process heavily driven by visuality can exhibit weaknesses undermining certain aspects of spatial experience despite the complexity. Grounded in Merleau-Ponty’s notion of multisensory perception, this article discusses the relationship between body awareness and spatial perception and its implication for design disciplines concerning built environments. Special attention is given to the concepts of kinesthetic and synesthetic phenomena known as multisensory and cross-sensory, respectively. This discussion integrates the corporeal and spatiotemporal realms of human experience into the discourse of kinesthetic and synesthetic perceptions. Based on the conceptual, theoretical, and precedent analyses, this article proposes three models for design thinking: Synesthetic Translation, Kinesthetic Resonance, and Kinesthetic Engagement. To discuss the concepts rooted in action-based perception and embodied cognition, this study borrows the neurological interpretation of haptic perception, interoception, and proprioception of space. This article suggests how consideration of the kinesthetic or synesthetic body can deepen and challenge the existing models of the perceptual aspects of environmental psychology adopted in design disciplines.

## Introduction

Spatial perception involves the tangible elements of the setting and the intangible attributes, including atmosphere and energy, and the cognitive process of the multimodal (or cross-modal in some cases) sensory input. The spatial experience is fundamentally bound to the body that is not a mere receptor of sensory stimuli but an active agent that engages with the perceivable environment and apprehends the experience in which the senses mediate the relationship between mind and body as well as idea and space (Mandik, 2005). While studies from various disciplines have shown the link between mind and body or thinking and acting (Schön, 1983; Durão, 2009; Wilde et al., 2011; Kwon, 2018; Sheets-Johnstone, 2019; Tversky, 2019), the term “design thinking” seems to disconnect conceptual thinking from physical expression or manifestation, which appears to echo body–mind dualism (Wilde et al., 2011; Loke and Robertson, 2013; Sheets-Johnstone, 2019; Domingo et al., 2021). The body is not separate from the mind, and the way the human being perceives space is interdependent on the physical structure of the body. In the domain of represented space that is apprehended through perceptual and sensorial mechanisms, mobility is the primary vector and provider of meaning (Durão, 2009, p. 399). Cognitive neuroscience studies have found the relationship between perception and motor action in aesthetic experience and creative productivity (Hurley, 1998; Torrents et al., 2013); the connection of users’ visuospatial experience with locomotive behavior (Hoogstad, 1990; Tversky, 2005). However, the movement and position of the body have not much been discussed in relation to creativity and design thinking while the human body has long been a popular subject in design education, research, and practice concerning anthropometrics, human factors, and ergonomics that aim to decrease human errors and increase productivity and safety in the utilitarian use of the products. Despite the generally accepted perspective design can help mediate one’s existing movement or change its movement patterns (Fogtmann et al., 2008, p. 91), there has been a lack of consideration of body movement as a sensory modality and the ground for the possibility of spatial experience (Farnell, 2012).

While design education and research have integrated the conceptual, metaphoric, and visual aspects of movement (e.g., sense of movement in visual repetition of the same shape) into the early phases of design process, studies have criticized that design disciplines have emphasized the visuality above the other sensory domains (Attfield, 2000; Garner, 2018; Sheets-Johnstone, 2019). They heavily engage with the expression and perception of visual elements (Pallasmaa, 2005), emphasizing the visual aspect of Gestalt principles, which does not adequately explain the corporeal and kinesthetic aspects of spatial perception. Ponzo et al. (2018) pointed out “the contribution of certain modalities, such as the vestibular system and interoception, to multisensory integration and body ownership has only recently been studied and hence remain poorly understood” (p. 312). Despite such concerns, the theory of design elements and principles posits the visual qualities as the primary aesthetics of design and understates the importance of the other senses. Although the visual approach may provide a practical means to represent and communicate ideas, a design process heavily driven by visual aspects can exhibit weaknesses undermining other aspects of the human environment. Thus, any theory that restricts perception to a particular modality fails to fully explain diverse sensory phenomena, especially in multidimensional space (Svanæs, 2013).

Another issue is that approaches to human perception sometimes seem overly analytical, the consequence of which is that the senses are often treated as if they worked independently from one another. Individuals’ perceptions and interpretations of their surroundings become highly multimodal upon occupying and experiencing a space (Pallasmaa, 2005; Dischinger, 2006; Franck and Lepori, 2007; Heylighen et al., 2009; Heylighen, 2011; Wastiels et al., 2013; Kwon and Kim, 2021). While the sensation is partial, the senses are distinct yet indiscernible; they are united through the body in becoming its perception, argued Merleau-Ponty (2014). Merleau-Ponty’s phenomenology of perception provides conceptual and theoretical insights into body, senses, and perception, as it concretes human existence, including subjective human experience, intentionality, action, perception, and meaning (Moran, 2000; Seamon, 2015). Crucial to the inquiry in environmental design is the phenomenological translation of the essence of one’s action and perception into architectonic dimensions, not only the examination of the impact of material elements on aesthetic or practical use. Due to the multidimensional, multimodal, and multisensory nature of spatial perception, no single methodology or prescriptive measure can sufficiently explain human responses to the spatial attributes and sensory stimuli (Budd, 2011). Regardless of some controversy, the reciprocal contribution of phenomenology and psychology to each other has been acknowledged for many years, and the contribution of phenomenology to environmental psychology and design has been noted as it provides insight into what one’s experience and perception are like for the subject from its first-person point of view (Seamon, 1982). In addition, there has been renewed interest in phenomenology research increasingly found in cognitive neuroscience, as researchers found the potential of phenomenology that can help bridge the gap between mind and brain (Albertazzi, 2021), which may also help explain the interrelationship body, mind, space, and time.

To further discuss the abovementioned issues, this article integrates the concepts of synesthesia and kinesthesia into the various discourses around the body and spatial perception and perspectives on body- and sensory-based design thinking. Poulsen and Thøgersen (2011) found the essence of design thinking as “reframing” through understanding and establishing concepts and meaning. This article will analyze and discuss how consideration of the kinesthetic or synesthetic body can challenge the existing models of perception adopted in design disciplines and deepen and enrich the way of design thinking and application. Finally, this article will propose new frameworks for design thinking that concern the body experiencing a space created.

## Perspective on Body, Senses, and Perception

The link between phenomenology, environmental psychology, and design has been addressed in various notions of architecture, body, the senses, and perception (Seamon, 1982, 2015; Pallasmaa, 2005; Zumthor, 2010). Phenomenology is the study of conscious experience, which has primarily concerned itself with phenomena of vision (Milner and Goodale, 1995, p. 13; Albertazzi, 2013, p. 5) and continuously inspired research on human experiences in various domains, especially perceptual experience and embodiment in environmental design disciplines. Phenomenology seeks the *essence* of lived experience, presupposing that human experience is *intentional*; our knowledge comes from what we experience; the essential meaning of our experience is hidden (Van Manen, 1997; Franzini, 2015; Seamon, 2015). Edmund Husserl is credited with initiating phenomenology as a discipline that seeks the essence of lived experience in the “lifeworld”—the day-to-day world where one’s ordinary pursuit takes place (Seamon, 2000).

The close relationship between phenomenology and built environments as well as other creative realms has been addressed by many phenomenologists, including Heidegger, Merleau-Ponty, Casey, Dewey, and Ihde. Particularly, the stance of existential phenomenology is that the lifeworld inevitably engages the body with the lived context. The construct of human existence comprises four existentials—spatiality, corporeality, temporality, and relationality (Merleau-Ponty, 2014)—in a communicative relationship with the lifeworld constructed of lived space, lived body, lived time, and lived others (Van Manen, 1997); the four existentials play an essential role in embodiment. Embodiment refers to the tangible or visible form of perceived concept and meaning through which ordinary life is incorporated into the body and becomes naturalized in the form of space (Attfield, 2000). An embodied space is imbued with one’s own memories, imaginations, and dreams accumulated through the personal and/or collective experience of the space, which fundamentally involves the body and movement, whether actual or conceptual (Cresswell, 2004). Considered “experience” is from an embodied position (Ihde, 2012). Lived experience occurs in the intersubjective space of perception and the body, located between subject and object (Simonsen, 2005; Merleau-Ponty, 2014). The lived human existence is a complex, multidimensional relationship and continuous dialogue with the external world and the self. In it, every essential experience and aesthetic judgment arises in connection with a contextual whole called “situation” (Dewey, 1998); thus, there are no inherent aesthetics of objects, buildings, and spaces. Phenomenologists argue that visual appearances of things are presented with meaning, given by their qualitative characteristics such as size, scale, proportion, and reciprocal positions; meaning is the content of experience, “not semantic content but rather the intuitive coherence things have for us when we find them and cope with them in our practical circumstances” (Carman, 2014, p. x); the meaning enhances the subject’s experience of the visual (Lu et al., 2011; Albertazzi, 2013).

The spatiality of the lived body is discussed in phenomenological discourses of embodied space: the personal, physical experience of space, *muscular consciousness* (Massey, 2006); spatial embodiment as “the form of inner sense [and] contains compressed time” (Casey, 1997, p. 289); a place to which one is emotionally attached, as a series of places with own memories, imaginings, and dreams (Bachelard, 1964; Cresswell, 2004). The phenomenological concept of embodiment does not account for a distinction between “being” and “having a body” and between “feeling” and “perceiving” (Sheets-Johnstone, 2019). Embodied space is not a mere collection of rooms and things but one’s embodied self that inhabited the space over time; space is incorporated into the body and can be naturalized in embodiment; thus, the embodied self is central to the lived space. Spatial experience is through sensing, the means and fundamental of being (Merleau-Ponty, 2014). It involves material practice in various modes, through which people conceptualize space and time and in which they apply the concepts (Harvey, 1989) contingent upon the lived state of one’s mind and body that occupy the space and perceive and act upon the setting (Bechtel and Churchman, 2003; Graumann, 2002; Pallasmaa, 2005). Together, the senses, mind, and body are integral to the total experience, so are ideas and objects.

Rooted in spatiotemporal and kinesthetic reality is the existence of the lived body comprised of continuous felt experiences, not simply its physical presence (Merleau-Ponty, 2014; Sheets-Johnstone, 2019). Merleau-Ponty’s phenomenology of perception (2014) emphasizes the subjective sensory processes of the lived body, “being a self of movement” or “feeling of doing,” tie the three aspects of lived body—felt, experienced, and sensed body. This work influenced proposal of Casey (1997) that the body is fundamental to place and exists in three modes—staying in, moving within, and moving between places. Merleau-Ponty also suggested that the body itself is expression that is simultaneously constituted with thought: like connotative language, the body is “a general system of symbols” that does not presuppose but rather accomplishes thought. For him, a human is a “sensorium commune” whose body accesses the world through the senses; perception of space is not a mere collection of perceptions of objects but a “flow of experiences” that expresses the spatiality of the human being.

One’s perception of the external world and its own body is based on “the integration of sensory information conveyed by different modalities each weighted according to their contextual reliability” (Ponzo et al., 2018, p. 311). Sensing is the experience of a modality of the body while the senses communicate through the body (Carman, 1999, 2014); while sensation is partial, the senses “distinct yet indiscernible, like monocular images in binocular vision” (Merleau-Ponty, 2014, p. 239), are united through the body forming a perception. Thus, neither sensing nor perception can fully be understood when the world is (mis)taken “as ready-made or as the milieu of every possible event and treats perception as one of these events” (Merleau-Ponty, 2014, p. 214).

### Action and Perception

In the traditional definitions, sensation, perception, and cognition are viewed as distinct phases in acquiring and processing information: the sensory organs gather stimuli in the sensation phase; in perception—the first phase in the thought process—the brain interprets sensations and organizes the information into patterns; the second phase of the thought process is cognition, “the way the information and knowledge come to be known, through the actions of perception, reasoning, or intuition” (Kopec, 2012, p. 51). Research has shown various perspectives on environmental perception. One of them is that visual perception is dominant when people acquire and process information from their surroundings: people derive as much of their perception of distance and movement from visual cues within a space despite sometimes conflicting non-visual cues (Axelrod, 1973; Harris et al., 2000; Kopec, 2012); consequently, they become less aware of movement within a space or senses responding to other corporeal aspects if there is an abundance of visual information (O’Regan and Noë, 2001; Hurley and Noë, 2003; Sun et al., 2004). More recent studies have stressed that action and perception attribute, in tandem, to making sense of the context and content of space: action and perception are embedded in each other and bound to one’s physical body and body awareness (Garner, 2018). One’s bodily states provide judgments and perceptions, and sensorimotor stimulations influence those judgments made (Brouillet et al., 2010; Ionta et al., 2011). Research on perception and cognition has adopted sensorimotor approaches (Hurley, 1998; Torrents et al., 2013), embracing the phenomenological concept of embodiment (Albertazzi, 2013, p. 5).

In the 18th century, the relationship between action and perception became an interest of philosophers and psychologists, including Berkeley. He initially proposed that vision was to be determined by visual depth cues, the movement of one’s eyes, with the adaptation of lens and when paired with touch would allow for people to move and interact with space and objects and therefore develop a “perception of the sensation” (Berkeley, 2008). In 20th century, action-based perception evolved from initially focusing on the movement of one’s eye to inform their spatial experience and perception and moved to be thought of as enactive: sight depends on one’s “sensory effects of movement” through a two-step process: users must experience the sensory stimuli and then use the sensory stimuli to retrieve sensorimotor contingencies associated with that object based on past experiences (O’Regan and Noë, 2001; Noë, 2010, p. 249). The concepts of embodiment and embodied cognition stress the mind (brain)–body connection in perception and cognition and gives attention to the impact of the interaction between the sensorimotor aspects of body and physical environments (Brouillet et al., 2010). Theories such as the motor component theory (Shebilske, 1984, 1987; Ebenholtz, 2002; Helmholtz, 2005) and the efferent readiness theories, modest readiness theory, and bold readiness theory (Coren, 1986) emerged stating that one’s ability to process stimulus information is optimized by the input of additional information to aid the visual information, specifically looking at proprioceptive feedback and actions such as turning around or turning upside down affect one’s understanding of the surrounding environment and objects within it. The embodied aspects of sensorimotor activities in human learning, knowing, and reasoning have been studied in education, including child learning and STEM education (Abrahamson and Bakker, 2016; Tversky, 2019). Linguistics brings to light the relationship between action and perception and linguistic responses that abstract concepts are grounded metaphorically in embodied and situated knowledge (Brouillet et al., 2010, p. 312). Studies in robotics and interactive product design also focus on somatosensory phenomena (Van Rompay and Ludden, 2015; Shima and Sato, 2017), as the action and perception of objects or space take a significant role in one’s experience (Noë and Noë, 2004; Brouillet et al., 2010).

### Kinesthetic Perception

Perception and cognition can be influenced by various factors such as type and intensity of stimuli, personal past experiences, current emotional state, or individuals’ physiological sensitivity. One’s perception of its environment, including objects, is in direct relation with its kinesthetic dimensions; the perception and the kinesthetic dimensions together create the meaning for the said environment or objects for the individual (Husserl, 1970; Gallagher and Zahavi, 2012). British neurologist Henry Charlton Bastian is credited with creating the term, kinesthesia. Kinesthesia is defined in various studies concerning bodily experiences: humans’ ability to sense one’s muscular movement from the lived body, the self-conscious subject perceiving its own body as the object experiencing and relating it to the environment or objects outside of the lived body encountered; the movements of the body and the kinetic sensations allow one to perceive and understand the space and objects within the environment it is inhabiting (Garner, 2018); a direct sensitivity to movement through internally mediated neuro-muscular systems (Sheets-Johnstone, 2019, p. 145). Humans possess a kinesthetic sense, affording them the ability to gain awareness of their body’s location and position in relation to their surroundings. Kinesthesia belongs to the lived body, as it represents the dynamism of embodied self-experience inside of the kinetic body (Garner, 2018, p. 146). The kinesthetic sense is beyond what they see, hear, and touch; it is a form of physical holistic (i.e., neurological transmission, motion, vision, and touch/tactile), aiding in an intuitive and instinctual recognition of the characteristic of a physical location. Kinesthetic intelligence is created through the lived body sensing movement expressed and experienced. Kinesthetic intelligence and awareness enable humans to better perceive the world and cope with it; by moving in the context, one can gain access to the meaning it has to the lived body (Melcón et al., 2017; Meglin et al., 2018; Korik et al., 2019). While Sheets-Johnstone (2019) defines kinesthesia as “the evolutionary descendant of proprioception” and Garner (2018) argues that, although similar in meaning, the term proprioception is often misused when conceptually describing kinesthesia: although kinesthesia may be explained with an emphasis on its proprioceptive aspects, it is not a favored term among the literature and certain disciplines concerning movement in spatial embodiment and perception.

**Figure 1:**
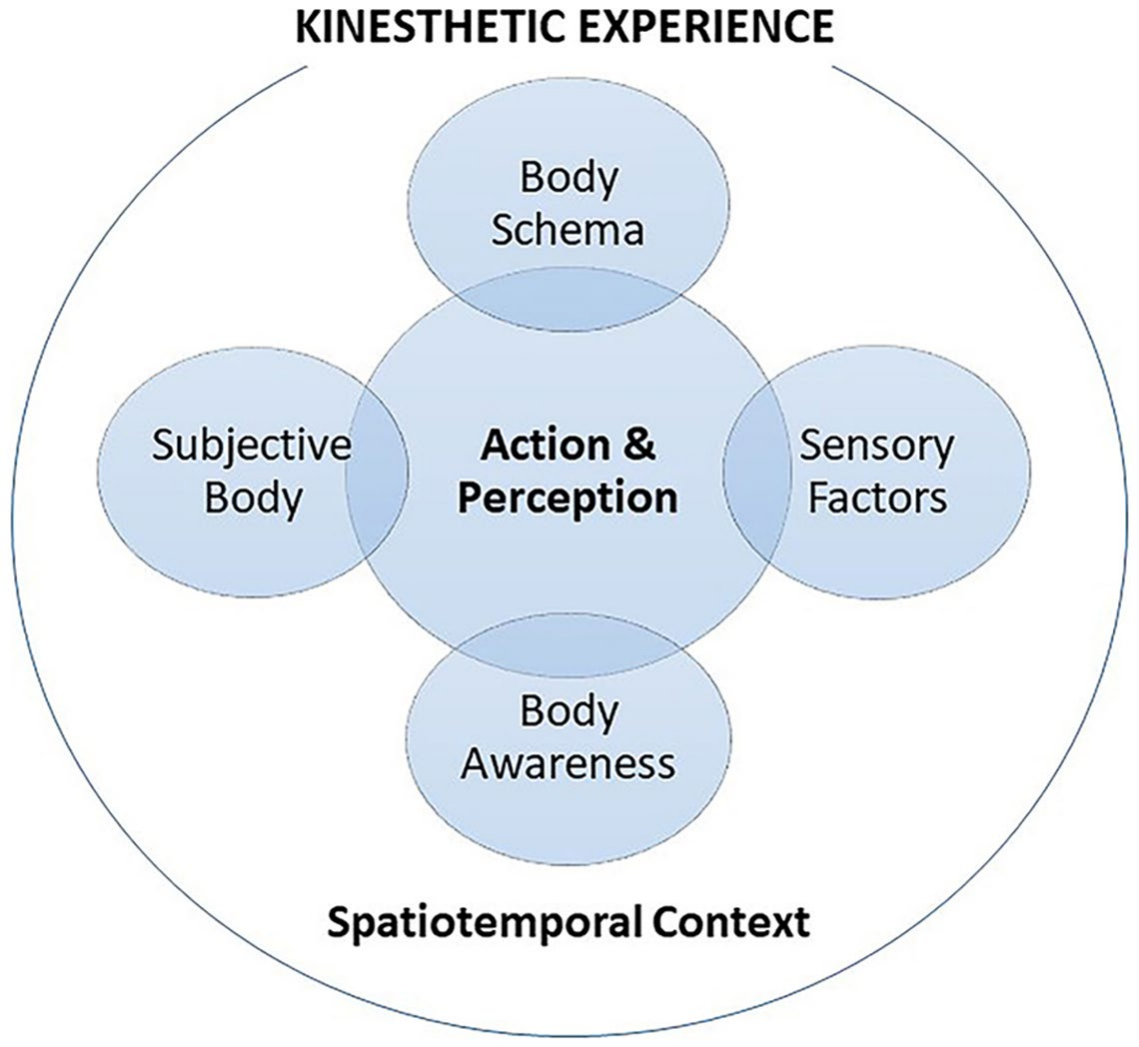
Construct of kinesthetic experience.

Kinesthetic experience is contextual and relational. In kinesthetic experience (Figure 1), the spatiotemporality of the lived body actualizes the articulation of sensory phenomena (Merleau-Ponty, 2014). For example, a tactile phenomenon disappears if any of the two, spatiality or temporality, is removed: “smoothness [or roughness] is the manner in which a surface makes use of the time of our tactile exploration or modulates the movement of our hand” (p. 329). As such, body movement involves tactile qualities that help individuals comprehend their surroundings. Insight into the senses in aesthetic experience of Franzini (2011) helps explain the invisible dimension of spatial experience:

The senses are essential to our understanding of form, but paradoxically the sensory form stands beyond what our senses can apprehend (p. 115) … [T]ouch is the sense that escapes isolation and opens to the totality of the aesthetic experience. It is an embodied perception, which goes beyond the clarity of “visibility” to include also the hidden power behind the apparent transparency of the representation. Touch indicates the possibility of reaping the hidden aspects of form, the invisible, the “unfinished” that…has been the response to the exclusively narrative, metaphorical or rhetorical view of art. In this way, one can affirm that touch is an ulterior method of opening the symbolic dimension of art, which is precluded…by its reduction to language or to only one of the senses. … [T]ouch…is bound to the ambiguity of a bodily gesture [and] is irreconcilable with any form of allegory or rhetoric (pp. 123–124).

Body movement (not a mere shift of locations or positions) in space is the foundation of one’s senses, and the kinematics of its movement is modified upon the relational context of the experience. The kinesthetic sense gives humans the ability to identify specific environmental characteristics and qualities and thus enhances the spatial experience (e.g., Cutts et al., 2019; Giroux et al., 2019). It is a high level of perception that involves the complex constitution of body schema, the representation of the body’s spatial properties, including exteroception, interoception, and proprioception (Valenzuela-Moguillansky et al., 2017).

**Figure 2:**
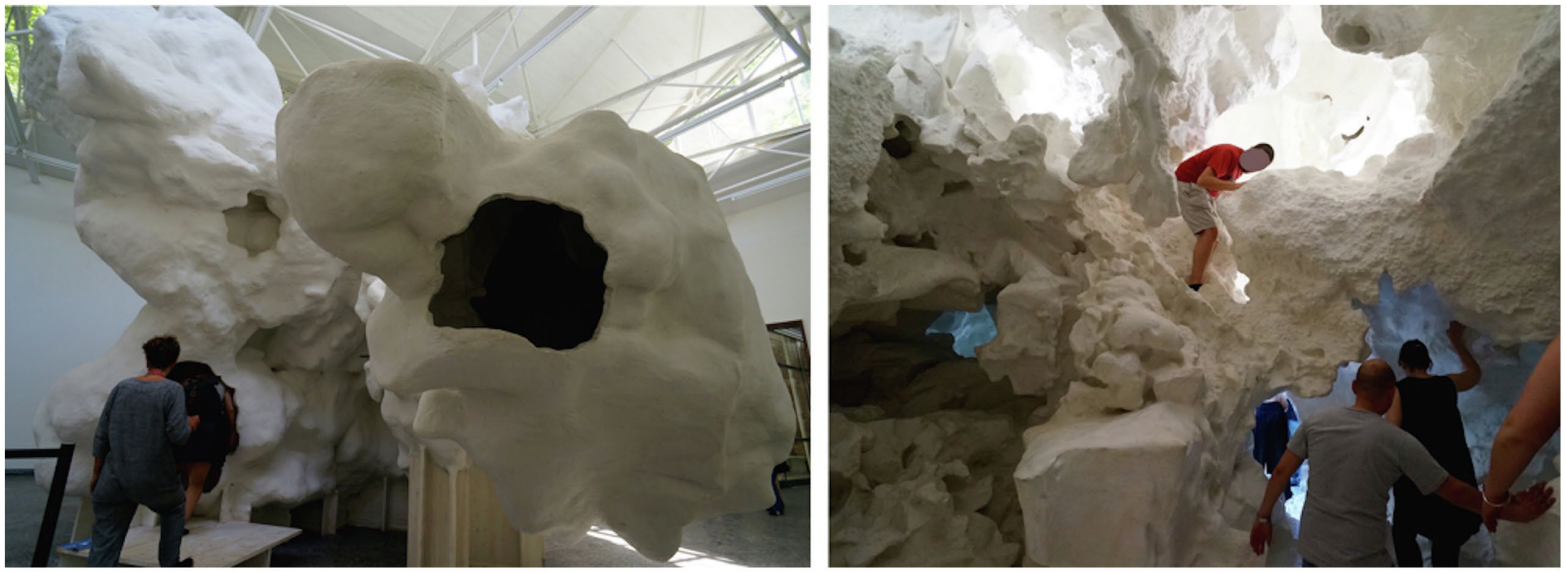
Pavilion *Incidental Space*, Christian Kerez, Venice Architecture Biennale 2016, Venice, Italy. Photo credit: Jain Kwon.

In the subjective human–environment dialogue, somatosensory factors such as orientation, position, temperature, texture, and pressure also play a significant role, impacting the felt body, conscious movement, bodily boundaries, and the peripersonal space (Pasqualini et al., 2013). The pavilion *Incidental Space*, designed by Christian Kerez, provides a distinctive kinesthetic experience that involves spatial awareness, positioning of one’s body, and/or perception of its movement acquired through physical sensations (Figure 2). The kinematics of individuals’ body movements responds to the spatial context: for example, as one attempts to reach higher than its height, passes through a narrow space, or passes by another person in close proximity. Such movements are also owing to the tactile and visual texture of the material as well as the sound and echo enhanced by the cave-like form of the inner space. These auditory, tactile, and visual factors together form the total experience of the space, contributing to the spatial identity and meaning visitors establish.

**Figure 3:**
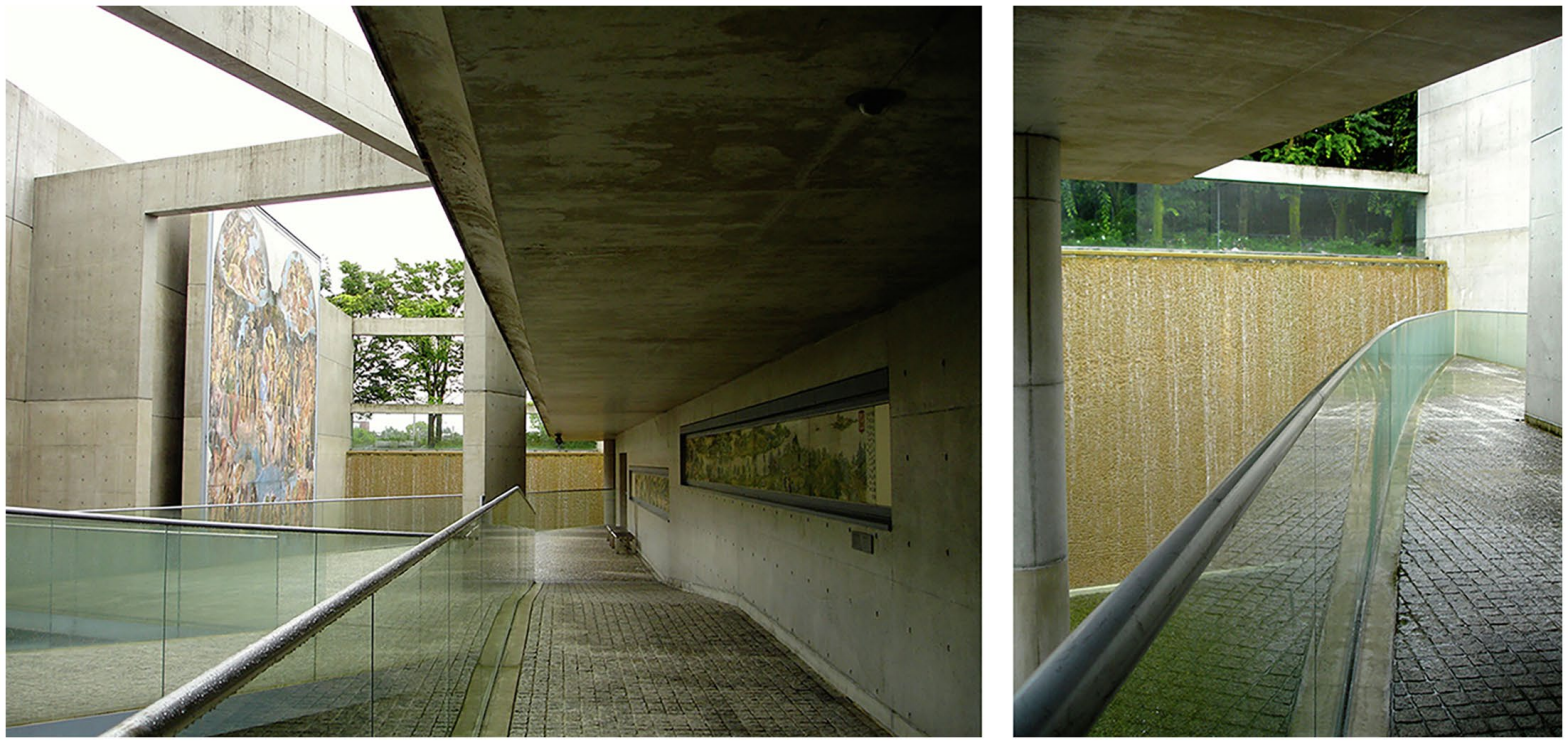
Outdoor gallery viewed from the lower level (left) and a cascade viewed from an elongated ramp (right). Garden of Fine Arts, Tadao Ando, Kyoto, Japan. Photo credit: Jain Kwon.

Spatiotemporality and kinesthetic perception are integral to each other. While Ando’s definition of space as “a place for many senses: sight, sound, touch, and the unaccountable things that happen in-between” points to the synesthetic dimension of sensory experience (Auping and Ando, 2002, p. 31), his work, including the Garden of Fine Arts (Figure 3) in Kyoto, Japan, often engages visitors in a kinesthetic journey throughout their experience in the settings. The spatial experience is enriched with the sensorial communication between the body and the environment: as one navigates the outdoor gallery, the sequence and gradual changes of the sound and the moist air from the cascades and the water features built around the elongated ramps comes into the total experience; the natural light and shadows change every moment while the navigating body communicates with the space in its motion and movement. No single moment is like another in the lived experience, and no single sense responds to the environment by itself; the spatiotemporality of the phenomena is the key to the total experience.

### Synesthetic Perception

**Figure 4:**
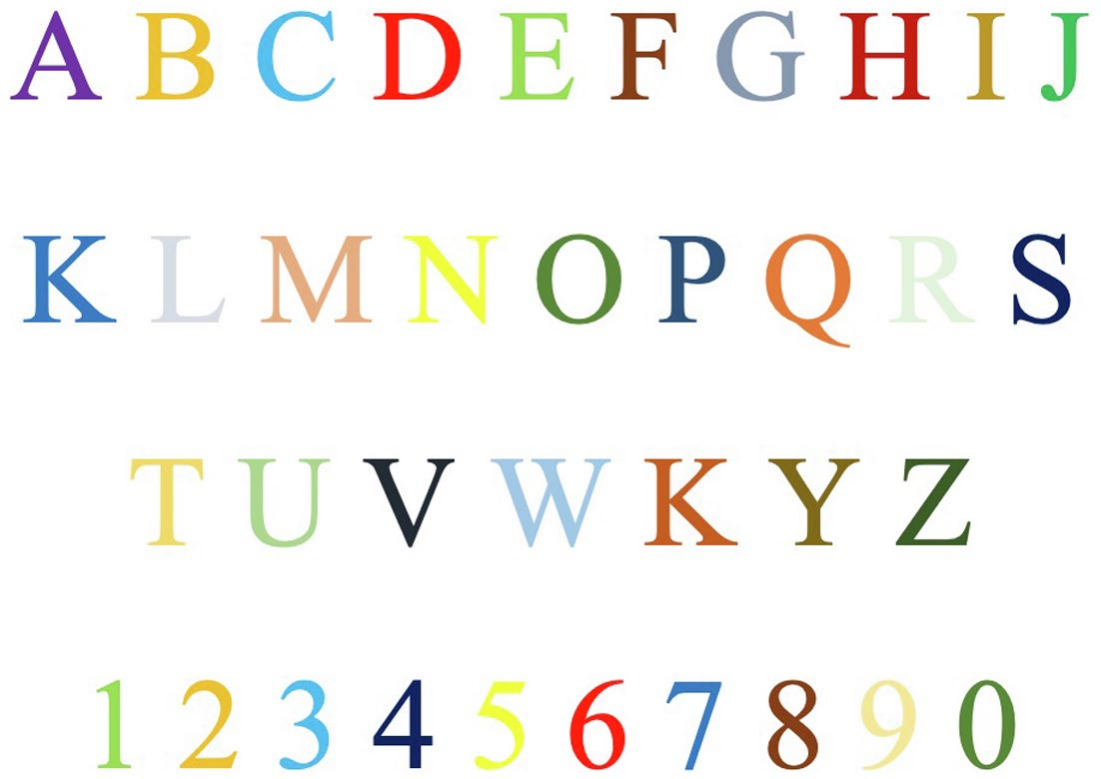
Example of a grapheme-color alphabet.

Synesthesia is a physio-psychological and cross-modal sensory phenomenon that is autonomous, involuntary, and irrepressible; it occurs when a stimulus in one sense modality immediately evokes sensations in one or more different sense modalities (Hubbard and Ramachandran, 2005; Van Campen, 2008; Merter, 2017). Synesthetes may see sounds, smell words, touch tastes, or taste letters, for example (van Leeuwen et al., 2016). When grapheme–color synesthetes see a number or a letter, they see a color at the same time (Figure 4), which is different from just imagining the color or making an association based upon memory (Ramachandran and Hubbard, 2003).

Due to the lack of information in the past, synesthesia was sometimes misunderstood as a neurological disorder, a brain impairment, or even a mental illness. However, there has been general appreciation for the synesthetic representation of artistic ideas found in many artists’ works, including that of Vincent van Gogh, Wassily Kandinsky, and Piet Mondrian (Ione and Tyler, 2003; Schneck et al., 2006; Van Campen, 2011; Melcher and Zampini, 2011). Kandinsky (1982) described his experience of listening to Wagner: “*I saw all my colors in my mind; they stood out before my eyes. Wild, almost crazy lines were sketched in front of me*” (p. 364). He also gave many of his painting’s musical titles, for example, *Compositions VII*, as if they were visible music. Nabokov (1989), in his *Speak, Memory*, described his grapheme–color synesthesia (e.g., Figure 4): “the long *a* of English alphabet […] has for me the tint of weathered wood, but a French *a* evokes polished ebony” (chapter 2, para. 2). The literature has also shown synesthetic metaphors such as “architecture as frozen music” by Goethe and “poetry of light” by Louis Kahn. The Renaissance architect Alberti described his synesthetic interpretation of architecture: “music and geometry are fundamentally one and the same; […] music is geometry translated into sound. […] In music, the very same harmonies are audible which inform the geometry of the building” (in Wittkower, 1971, p. 9). Synesthetic metaphors have also appeared in design research on human experience even though the studies do not explicitly address synesthesia, the sensory phenomenon. For example, whether intended or inadvertent, the semantic differential of connotative terms used in qualitative studies in the field often has synesthetic implications: tactile terms (e.g., hot–cold, rough–soft, and heavy–light) are used as semantic differential scale anchors to measure participants’ responses to visual stimuli; ambivalent terms (e.g., light, soft, high, dull, and sharp) are used for representing various sensory ideas such as visual, tactile, and aural (Madden et al., 2000; Yoon, 2008; Kwon and Kim, 2021).

The phenomenological perspective on subjective sensory experiences is explained often with reference to *quale* (plural *qualia*): a consciousness like an introspectively accessible “region” where variable modalities of sensing take place and, together, come into perception (Van Campen, 2008). However, Merleau-Ponty (2014) pointed out the traditional concept of *quale* (plural “qualia”) does not properly explain certain sensory phenomena such as synesthesia:

… synesthesia [cannot be explained, for example,] if vision is defined by the visual *quale*, or sound by the sonorous *quale* … [It is not] merely that has a sound and a color at the same time: it is the sound itself that [one] sees, at the place where colors form. This formulation is literally rendered meaningless if vision is defined by the visual quale, or sound by the sonorous quale. But it falls to us to construct our definitions in such a way as to find a sense for this experience, since the vision of sound and the hearing of colors exist as phenomena. … if we do not notice [synesthesia], this is because scientific knowledge displaces experience and we have unlearned seeing, hearing, and sensing in general in order to deduce what we ought to see, hear, or sense from our bodily organization and from the world as it is conceived by the physicist (pp. 237–238).

Merleau-Ponty’s stance on synesthetic perception is that human perception unites all sensory experiences into a single lifeworld, and thus the “total experience” of things is through our embodied senses:

The vision of sounds or the hearing of colors comes about in the same way as the unity of the gaze through the two eyes, insofar as my body is not a sum of juxtaposed organs, but a synergetic system of which all of the functions are taken up and tied together in the general movement of being in the world (Merleau-Ponty, 2014, p. 243).

Interpretation of Chumley (2017) of *quale* may support Merleau-Ponty’s stance by referring to sensing of intangible existence such as “energy” normally perceived by its relation to actualized objects across multiple sensory modalities—audible, smellable, tangible, tasteable, and visible; thus, quale needs to be viewed as what makes sense of our understanding of language or signs, which is constantly reconstructed and evolving, not as a stable system. In the same vein, Franzini (2011) suggests that “the specificity of the [senses] involved in the act of perception is always within a communicative context in which synesthetic perception is the rule” (p. 125). As the cross-modality of synesthesia has increasingly been discovered, studies have re-conceptualized and redefined synesthesia and proposed alternatives severing the exclusively sensory interpretation of synesthesia: synesthesia is a semantically induced phenomenon that involves high-level cognitive representation (Ward et al., 2007; Mroczko-Wąsowicz and Nikolić, 2014). Such propositions may encourage reconsideration of the traditional distinction between perception and cognition assumed for a long time in philosophy, psychology, and cognitive science (Mroczko-Wąsowicz and Nikolić, 2014).

Synesthesia is certainly not a skill or knowledge (to be figured out, so to speak) nor what everybody experiences. There have been attempts to conceptualize synesthesia in an easier way by determining the construct of the unique phenomenon: for example, synesthesia consists of a perceptive phenomenon, metaphor, and representation; features such as color or sound (qualitative) relate to subjective values; features such as image size or sound intensity (quantitative) relate to intersubjective values (Riccò et al., 2003). Such categorization may need careful interpretation, as some readers might misunderstand it as if synesthesia is some type of sensory association or imagination. From a designer’s perspective, the interpretations of synesthesia in the literature—for example, a secret sense, the sixth sense (Sherrington, 1906), a hidden sense, or “everyday fantasia” (Van Campen, 2008)—have an important implication: understanding the cross-modality of the senses may help designers establish a new mode of creative thinking and diverse perspectives on sensory phenomena and spatial experiences.

## Body and the Senses in Design Thinking

Design thinking methods in which designers’ empathy plays a role have encouraged the processes of understanding others (i.e., occupants or users), which is a matter of interpretation of mind and body (Plank et al., 2021) and attention to verbal and non-verbal, visual and non-visual, or tangible and intangible cues within the context. Kinesthetic and synesthetic concepts are not always clearly distinguishable from each other. Movement by (and through) the mindful body is foundational to our understandings of human experience (Sheets-Johnstone, 2019, p. 25). The mindful body is kinesthetically informed and can be synesthetically conceptualized—as no single sense can work by itself separately from the others. Because the mindful body is contextual and relational, individuals perceive and conceive space differently, which is affected by their own life experiences (Cialone et al., 2017) and other people in direct or indirect interaction with them. On the one hand, interior designers’ life experiences help them establish strong insights into design decisions and the design process. On the other hand, those experiences might dominate their conceptions of human experience and result in them relying on their self-reflection overlooking the perspectives of interior occupants despite that it is one of the most critical and challenging tasks of designers.

**Figure 5:**
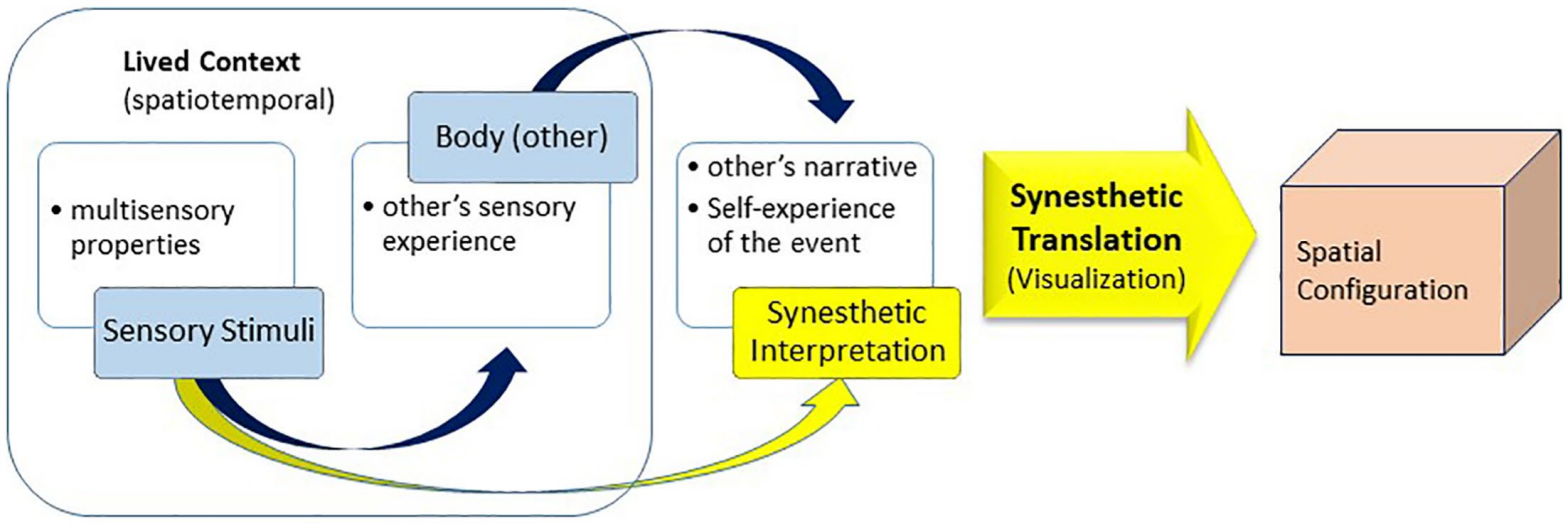
Synesthetic translation model for design thinking.

**Figure 6:**
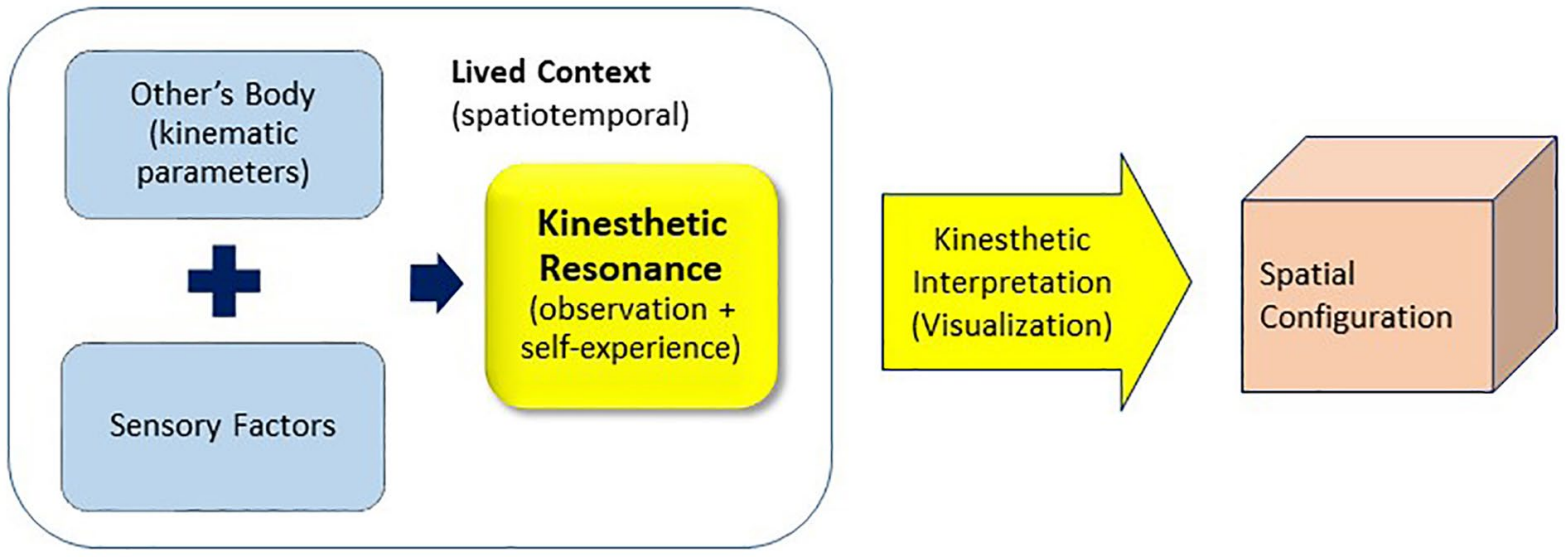
Kinesthetic resonance model for design thinking.

**Figure 7:**
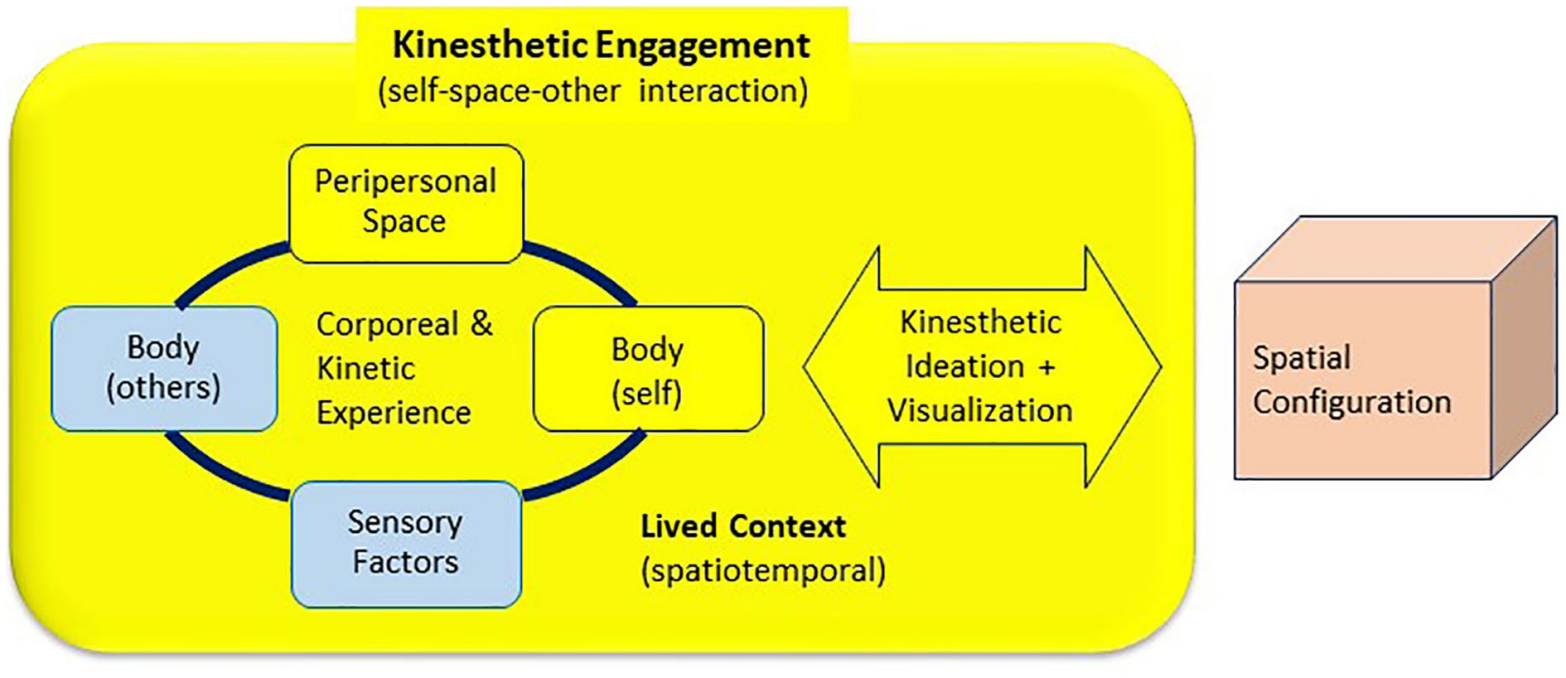
Kinesthetic engagement model for design thinking.

This paper proposes three design thinking models, suggesting that the design exploration incorporating the concepts of kinesthetic and synesthetic perceptions can foster diverse perspectives on occupant spatial experiences resonating with the environments. The cross-sensory concept of synesthesia and the corporeal and spatiotemporal aspects of kinesthesia are integrated into the three models for design thinking: synesthetic translation (Figure 5), kinesthetic resonance (Figure 6), and kinesthetic engagement (Figure 7). The processes of the three models include designer (*self*) and participants (*other*) in synesthetic or kinesthetic experiments to varying degrees. The three approaches were developed as pedagogical frameworks for an entry-level interior design studio course focused on collaborative design thinking and processes engaging participants with no design background.

### Synesthetic Translation Model

Visualizing abstract ideas and transferring them into spatial configuration is a conceptually translational process. The Synesthetic Translation Model (Figure 5) involves a participant’s (*other*) narrative of an auditory (e.g., musical) experience, designers’ (*self*) synesthetic interpretation of the non-visual and intangible sensory properties and qualities the participant describes, and the designers’ self-experience and reflection of the interactive context and event with the individual. The *self* and *other* can be single persons or groups depending on the project. This model shares some aspects of narrative design methods. While narrative methods have often been used to promote designers’ imagination in design thinking, the research raised a concern that using narrative methods may interfere with the visual representation of their imaginations and ideas (Danko et al., 2006). It is important to note that synesthetic phenomena are fundamentally lived, so they differ from imaginations or memory-based sensory associations. The design approach must concern the lived nature of occupant experiences. The synesthetic translation model emphasizes designers’ empathetic approach as reflection—not imagination—of participants’ lived experience.

An example of synesthetic translation design thinking uses music as a non-visual inspiration (*sensory stimuli*) and involves a participant representing *the body* (*other*) as a subject of sensory experience as well as an object the designers perceive in the interactive event and the spatiotemporal context. This approach focuses on the audiovisual and temporal realms of the participant’s musical experience and designers’ synesthetic interpretation of the participant’s narrative in the lived context and “translation” of the verbal description into a spatial setting. The synesthetic translation approach consists of five phases: (1) music plays as a sensory stimulus, (2) music replays during the participant’s concurrent think-aloud (narrative), (3) designers’ interpretation of the participant narrative, (4) visualization of the essences of participant experience, and (5) spatial configuration and prototyping. The participant’s concurrent narrative in this process can provide “vivid” descriptions of the lived experience, possibly implying the concept of multisensory and cross-sensory phenomena. Although music is typically described as an auditory phenomenon, it is, in fact, multimodal. For example, music engages the body with its vibrations, volumes, and cadences that rise and fall, increase and decrease, and quicken and slow (Garner, 2018, p. 172). The properties of sound (music), for example, intensity, volume, pitch, and rhythm, are closely linked to the concepts of spatial attributes such as compression/expansion, volume, scale, and pattern. Indeed, they are described in similar words, for example, heavy–light, strong–soft, rough–smooth, and dark–bright. Musical experience needs to be explained in spatial terms because music is the sounds ordered in time, which moves through the imaginary space of music (Scruton, 2004). The concept of movement is metaphorically applied to creating the sequence of space, in which designers’ interpretation of sensory experience and “synesthetic intelligence” play the key role. Due to the attention to the temporality of musical experience that is fundamentally sequential, the designs produced applying the synesthetic translation model tend to show linear (or spiral) progress or “journey” in the spatial configurations (Kwon, 2017, p. 390). Thus, this model may be adopted in design processes where storytelling is the key.

### Kinesthetic Resonance Model

Humans recognize and respond to their surroundings and other entities in the context, including others’ bodies and their kinematic parameters (Torrents et al., 2013; Garner, 2018). Individuals use their knowledge of their bodies and current and previous situations to understand abstract concepts (Lakoff and Johnson, 1999). The Kinesthetic Resonance Model (Figure 6) integrates the kinematic parameters of others (e.g., inspirational art performers, prospective occupants/users, and passers-by) observed by an audience (i.e., designers) into design thinking. Kinesthetic resonance refers to “the perceiving subject’s vicarious engagement with the movements of others”; the responses are situational, multi-directional, and variable (Garner, 2018, p. 145). In the perception of another’s intentional action, what we know about movement has an impact on the sense of engagement we experience and the vicarious engagement we feel in our muscles (Garner, 2018, p. 158). Empirical studies in performing arts have shown the relationship between kinesthetic and expressive qualities that reveal the emotion represented in the work of arts (Montero, 2012; Garner, 2018). Certain kinematic parameters in dance influence a non-expert audience’s aesthetic perception of the artistic expressions of movement (Torrents et al., 2013, p. 457). In one’s aesthetic experience in a constructed environment, empathy plays a role in its resonance with the space and as activation of embodied mechanisms (Freedberg and Gallese, 2007; Jelić et al., 2016).

Attfield (2000) found dance and music as useful tools for explaining a sense of movement in relation to space and time: innately, dance, music, and space are present in time, which is a channel through which human existence represents a sense of temporality and continuity. Seamon (1980) has used the metaphor of dance and “time–space routines” to characterize the sequences of actions that make up everyday practices (Cresswell, 2004). Thus, dance can be used as an inspirational tool in design exploration based on designers’ observation of movements (e.g., amplitude, turning velocity, balance duration, jump height, and range of motion) and their experience of the event in relation to the spatial setting and other circumstances. Dancers’ movements not only show the postures and positions of their bodies but also convey the dynamics that affect the audience’s kinesthetic resonance: kinetic energy and human–human and human–environment interactions—for example, dancer–dancer, dancers–audience, dancers–space, dancers–music, and audience–music—in the space and time.

The kinesthetic resonance approach illustrated in Figure 6 is inspired by (not direct reflection of) the phenomenological concept of John Cage’s composition 4′ 33″ that uses an “expressive silence” as a means of engaging the audience in the abstract dialogue during the piano performance: while a pianist is sitting at the piano for 4 min and 33 s, no piano music is played. Some people might view the “performance” as plain silence beside the random sounds the audience makes (e.g., rustling and creaking noises from people shifting in their seats and coughing) because the performance does not convey a particular musical intention. Yet, the pianist still “performs,” creating the “expressive silence” that embraces the unpredictable and lived event (Cage and Gann, 2011). The audience is contributing to the performance by being part of the lived context, observing the performers (and perhaps the others in the audience), and reacting to the circumstance in which the audience’s kinesthetic resonance takes place.

When the kinesthetic resonance approach is adopted in exploratory design projects engaging professional dancers and music, it often results in outcomes that the envelope—rather than the space inside—of the designed space tends to be representational of the concept and resemble the visual of the dancers’ body postures in a captured moment or rotational movement. It may be because the approach heavily relies on the designers’ observation. This model may better suit the conceptual visualization of an observed scene or designing an object or relatively small structure (e.g., fixture, pop-up kiosk, and three-dimensional artwork) that the view from the outside is one of the primary interests in the design process.

### Kinesthetic Engagement Model

Spatial perception is reciprocal with self-consciousness: the sense of agency, sense of body ownership, and self-location (Longo et al., 2008; Pasqualini et al., 2013; Galvan Debarba et al., 2017). In other words, spatial perception occurs through the embodiment of the material properties of the environment (Gibson, 1979; Blanke and Metzinger, 2009); the sense of embodiment emerges from the feeling of motor control over one’s own body perceived in its location. The content of spatial experience is enacted by action engaging the body and its sensory mechanism (Noë and Noë, 2004), to which kinesthetic perception is key.

Corporeal concepts originate in the context of moving (action) and thinking in movement (Sheets-Johnstone, 2019). Embodied design approach foregrounding the kinesthetic sense is an important design strategy in which how moving and knowing bodies can impact the way designers think and work (Wilde et al., 2011; Loke and Robertson, 2013; Kwon, 2018, 2020). The Kinesthetic Engagement Model (Figure 7) represents action-based design thinking and the embodied processes through human–human and human–space interactions. The interactions are enacted by the actors—the self (designers) and others—engaged in bodily movements and conceptualizing the movements in relation to the space and time in which the movements take place.

**Figure 8:**
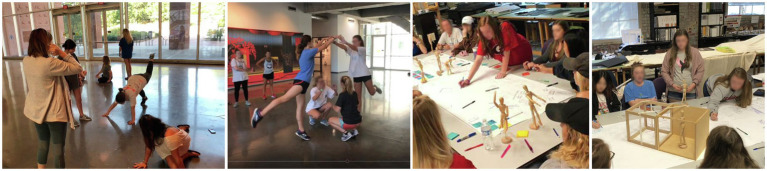
Kinesthetic design process engaging the body: bodily experiment and ideation. Photo credit: Jain Kwon.

**Figure 9:**
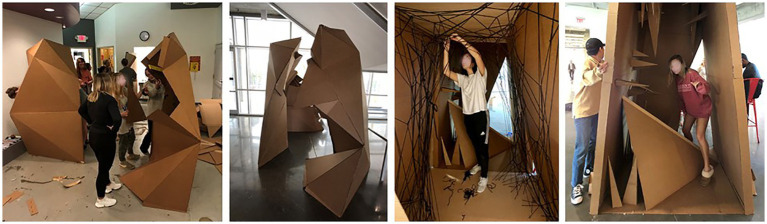
Full-scale prototyping. Photo credit: Jain Kwon.

Movement-based approaches can foster the connection with emotion and bodily sensations in sensorimotor processing, establishing coherent body awareness and gaining familiarity with bodily sensations as part of embodied subjectivity (Valenzuela-Moguillansky et al., 2017). The project illustrated in Figures 8, 9 used the kinesthetic engagement model focused on occupant bodily engagement and experience in a setting. The design process included experiments (Figure 8) focused on how physical bodies—the self’s and other’s—could create the sense of space, territory, or boundary, responding to the surrounding. For the project, interior design students played dual roles, occupants (users) and designers, to learn occupant experience from the first-person point of view and incorporate it in their designs. Students “choreographed” the dialogue between their own bodies and space, conceptualizing the gestures, movements, positions, and postures: for example, balance, stability, tension, fluidity, and containment often discussed in design disciplines. This experiment was followed by ideation conveying the concepts of the body and movement in a confined space (Figure 8). Finally, students designed and built full-scale structures of experiential space (Figure 9), portraying their sensorimotor and somatosensory experiences through their bodily exploration.

One’s kinesthetic intelligence is affected by the felt scale of its own body and relationship with the spatial setting. Prototyping (except for study mock-up) in the kinesthetic engagement approach was conducted on a 1:1 scale. The outcomes through the three approaches—synesthetic translation, kinesthetic resonance, and kinesthetic engagement—with designers’ bodily engagement to varying degrees, showed interesting patterns in design outcomes (Table 1): (1) designs using the synesthetic translation model presented sequential order of space; (2) many outcomes through the kinesthetic resonance approach appeared in spiral configuration and the exterior form reflected body posture captured in a specific moment of the total movement; (3) designs through the kinesthetic engagement processes showed their emphases on the interior configuration and space, the negative form of which resembled and evoked various body movements.

**Table 1:** Construct of three design thinking models: synesthetic translation, kinesthetic resonance, and kinesthetic engagement.

	Synesthetic translation	Kinesthetic resonance	Kinesthetic engagement
Perceptual Emphasis	Synesthetic (cross-modal)	Kinematic	Kinesthetic
Sensory Stimuli	Auditory: Music	Auditory + Visual: Music & Dance	Auditory + Visual + Haptic: Bodily Experiment
Perspective on occupant experience	Reflective	Observational	Experiential
Emphases in design approach	Empathy in Aesthetic Experience Understanding Others’ Sensory Experience	Embodied Space Observation and Interpretation of Movement in Spatiotemporal Context	Embodied Design Process through Bodily Engagement
Designer’s key role in experiment	Interpreter	Observer & Interpreter	Actor
Design emphasis	Descriptive reflection of other’s narrative	Observation and descriptive reflection of human–human and human–space interactions	Use of own body to determine forms and the scale in ideation
Tendencies found in design outcomes	Representative of a “journey”: linear configurations & perpendicularly sequential order of occupant experience Designed space in “monument” scale Reflection of emotional feelings	Reflection of captured moments and the scenes: circular, spiral, curvilinear configurations Vertically sequential order of occupant experience Designed space in human scale Visual description of the kinematic parameters of others’ bodies	Reflection of body movements, postures, bodily interactions with others, and physical settings Non-linear; multiaxial Designed space in human scale Description of body movement and kinesthetic responses

## Discussion

Based on the conceptual and theoretical analysis of synesthetic and kinesthetic perceptions, this article proposed three design thinking models: the synesthetic translation and kinesthetic resonance models are based on designers’ descriptive reflection through listening and observing; the kinesthetic engagement model emphasizes designers’ bodily engagement and interaction with others and the space. Engaging the body, senses, and movement in design thinking can help determine the relationship between the designed environments and the end-users. Design thinking is fundamentally embodied and, like perception, innately lived; it differs from memory-oriented associations or imaginations in which the body is dislocated from time and space (Hubbard and Ramachandran, 2005). Spatial experience involves the subjective, multimodal, and contextual body and sensorimotor phenomena; bound in time is an active (non-linear) process of establishing meaning based on one’s awareness and understanding of the self, others, and its surroundings. Embodied design approach that is requisite for the creation of meaningful space cannot be reduced to a linear process of ideation, analysis, and synthesis clearly separated. Designing is an embodied process through being a self of movement and feeling of doing, which is perceptual, perceptive, and expressive. The concepts of body awareness, sensory experience, and spatial perception are increasingly diversified, especially with emerging technologies, including mixed reality and motion-sensing. Constructed—whether physical or virtual—environments are experienced through the presence of its occupant being a self of movement or feeling of doing that ties felt/feeling, experienced/experiencing, and sensed/sensing body together. Some of the sensory responses of the body are seldom integrated into design thinking and yet to be explored, for example, synesthetic and phantom sensations—caused by immersion in VR environments.

This article suggests that abstract conceptualization and bodily engagement are not entirely separate processes in design and stresses that spatial perception is fundamentally experiential and lived, neither imaginary nor assumptive. Design approaches that integrate kinesthetic and synesthetic experiences and perceptions anchored in the lived body can help enhance designers’ understanding and incorporation of aesthetic sensibility—how people perceive and appreciate sensory phenomena—and mind–body connectivity in design thinking. Design disciplines may reexamine the traditional concepts of perception and cognition as separate phases of information processing through the sensory system. This article discussed how consideration of the kinesthetic or synesthetic body and phenomena could deepen and challenge the existing models of human perception and aspects of environmental psychology adopted in design disciplines. Looking into the integration of tangibility and intangibility into design thinking, and the strengths and weaknesses of experiential, observational, and imaginary approaches that have been adopted in design thinking may also provide provocative new insights into what the body means for designers to consider, and such efforts can contribute to the body of knowledge in environmental design disciplines.

## Author Contributions

JK developed the study, conducted the conceptual and theoretical analysis, developed the proposed models, and wrote the manuscript. AI, a graduate research assistant, assisted with the literature review. All authors contributed to the article and approved the submitted version.

## Conflict of Interest

The authors declare that the research was conducted in the absence of any commercial or financial relationships that could be construed as a potential conflict of interest.

## Publisher’s Note

All claims expressed in this article are solely those of the authors and do not necessarily represent those of their affiliated organizations, or those of the publisher, the editors and the reviewers. Any product that may be evaluated in this article, or claim that may be made by its manufacturer, is not guaranteed or endorsed by the publisher.

## References

[ref1] Abrahamson D. Bakker A. (2016). Making sense of movement in embodied design for mathematics learning. Cogn. Res. Princ. Implic. 1:33. doi: 10.1186/s41235-016-0034-3, PMID: 28180183 PMC5256464

[ref2] Albertazzi L. (2013). Handbook of Experimental Phenomenology: Visual Perception of Shape, Space and Appearance. John Wiley & Sons.

[ref3] Albertazzi L. (2021). Experimental phenomenology: what it is and what it is not. Synthese 198, 2191–2212. doi: 10.1007/s11229-019-02209-6

[ref4] Attfield J. (2000). Wild Things: The Material Culture of Everyday Life. Oxford: Berg.

[ref5] Auping M. Ando T. (2002). *Seven Interviews with Tadao Ando*; [published in *Conjunction with the Opening of the New Modern art Museum of Fort Worth, Designed by Tadao Ando, December 2002*]. Modern Art Museum of Fort Worth.

[ref6] Axelrod R. (1973). Schema theory: an information processing model of perception and cognition. Am. Polit. Sci. Rev. 67, 1248–1266. doi: 10.2307/1956546

[ref7] Bachelard G. (1964). *The Poetics of Space*. (M. Jolas, Trans.; new foreword by J. R. Stilgoe). Boston, MA: Beacon Press. (Original work published 1958).

[ref8] Bechtel R. B. Churchman A. (2003). Handbook of Environmental Psychology (1 Aufl.). New York, NY: Wiley.

[ref9] Berkeley G. (2008). “An essay towards a new theory of vision,” in Berkeley: Philosophical Writings. ed. Clarke D. (Cambridge: Cambridge University Press).

[ref10] Blanke O. Metzinger T. (2009). Full-body illusions and minimal phenomenal selfhood. Trends Cogn. Sci. 13, 7–13. doi: 10.1016/j.tics.2008.10.003, PMID: 19058991

[ref11] Brouillet T. Heurley L. Martin S. Brouillet D. (2010). The embodied cognition theory and the motor component of “yes” and “no” verbal responses. Acta Psychol. 134, 310–317. doi: 10.1016/j.actpsy.2010.03.003, PMID: 20394911

[ref12] Budd C. (2011). Valuing the intuitive: reintroducing design into interior design education. J. Inter. Des. 36, v–xi. doi: 10.1111/j.1939-1668.2011.01059.x

[ref13] Cage J. Gann K. (2011). Silence: Lectures and Writings, 50th Anniversary Edition. Middletown, CT: Wesleyan University Press.

[ref14] Carman T. (1999). The body in Husserl and Merleau-Ponty. Philos. Top. 27, 205–226. doi: 10.5840/philtopics199927210

[ref15] Carman T. (2014). “Forward,” in Phenomenology of Perception. ed. Merleau-Ponty M. (Abingdon, Oxon, NY: Routledge), vii–xvi.

[ref16] Casey E. (1997). The Fate of Place: A Philosophical History. Berkeley, CA: University of California Press.

[ref17] Chumley L. (2017). Qualia and ontology: language, semiotics, and materiality; an introduction. Signs Soc. 5, S1–S20. doi: 10.1086/690190

[ref18] Cialone C. Tenbrink T. Spiers H. J. (2017). Sculptors, architects, and painters conceive of depicted spaces differently. Cogn. Sci. 42, 524–553. doi: 10.1111/cogs.12510, PMID: 28656679 PMC5873447

[ref19] Coren S. (1986). An efferent component in the visual perception of direction and extent. Psychol. Rev. 93, 391–410. 3774917

[ref20] Cresswell T. (2004). Place: A Short Introduction. Malden, MA: Blackwell.

[ref21] Cutts S. A. Fragaszy D. M. Mangalam M. (2019). Consistent inter-individual differences in susceptibility to bodily illusions. Conscious. Cogn. 76:102826. doi: 10.1016/j.concog.2019.102826, PMID: 31670011

[ref22] Danko S. Meneely J. Portillo M. (2006). Humanizing design through narrative inquiry. J. Inter. Des. 31, 10–28. doi: 10.1111/j.1939-1668.2005.tb00408.x

[ref23] Dewey J. (1998). The Essential Dewey: Pragmatism, Education, Democracy, *Vol*. 1. Bloomington, IN: Indiana University Press.

[ref24] Dischinger M. (2006). “The non-careful sight,” in Blindness and the Multi-Sensorial City. eds. Devlieger P. Renders F. Froyen H. Wildiers K. (Antwerp, Belgium: Garant), 143–176.

[ref25] Domingo L. Gutzeit M. Leifer L. Auernhammer J. (2021). “Contemporary issues in remote design collaboration,” in Design Thinking Research. Understanding Innovation. eds. Meinel C. Leifer L. (Cham: Springer).

[ref26] Durão M. J. (2009). Embodied space: a sensorial approach to spatial experience. AIP Conf. Proc. 1103, 399–406. doi: 10.1063/1.3115544

[ref27] Ebenholtz S. (2002). Oculomotor Systems and Perception. Cambridge: Cambridge University Press.

[ref28] Farnell B. (2012). Dynamic Embodiment for Social Theory. New York, NY: Routledge.

[ref29] Fogtmann M. H. Fritsch J. Kortbek K. J. (2008). “Kinesthetic interaction: revealing the bodily potential in interaction design.” in *Proceedings of the 20th Australasian Conference on Computer-Human Interaction*, December 8-12, 2008; 89–96.

[ref30] Franck K. A. Lepori B. (2007). Architecture from the Inside Out. London, UK: Wiley.

[ref31] Franzini E. (2011). “Rendering the sensory world semantic,” in Art and the Senses. eds. Bacci F. Melcher D. (Oxford, UK: Oxford University Press), 115–148.

[ref32] Franzini E. (2015). Phenomenology and neuroaesthetics. Aisthesis 8, 135–145. doi: 10.13128/Aisthesis-16212

[ref33] Freedberg D. Gallese V. (2007). Motion, emotion and empathy in esthetic experience. Trends Cogn. Sci. 11, 197–203. doi: 10.1016/j.tics.2007.02.003, PMID: 17347026

[ref34] Gallagher S. Zahavi D. (2012). The Phenomenological Mind. 2nd Edn. London: Routledge.

[ref35] Galvan Debarba H. Bovet S. Salomon R. Blanke O. Herbelin B. Boulic R. (2017). Characterizing first and third person viewpoints and their alternation for embodied interaction in virtual reality. PLoS One 12:e0190109. doi: 10.1371/journal.pone.0190109, PMID: 29281736 PMC5744958

[ref36] Garner S. B. (ed.) (2018). Kinesthetic Spectatorship in the Theatre. Cognitive Studies in Literature and Performance (Cham: Palgrave Macmillan), 145–183.

[ref37] Gibson J. J. (1979). The Ecological Approach to Visual Perception. Boston, MA: Houghton Mifflin.

[ref38] Giroux M. Barra J. Barraud P. A. Graff C. Guerraz M. (2019). From embodiment of a point-light display in virtual reality to perception of one’s own movements. Neuroscience 416, 30–40. doi: 10.1016/j.neuroscience.2019.07.043, PMID: 31377453

[ref39] Graumann C. F. (2002). “The phenomenological approach to people-environment studies,” in Handbook of Environmental Psychology. eds. Bechtel R. B. Churchman A. (Hoboken, NJ: John Wiley & Sons), 95–113.

[ref40] Harris L. R. Jenkin M. Zikovitz D. C. (2000). Visual and non-visual cues in the perception of linear self motion. Exp. Brain Res. 135, 12–21. doi: 10.1007/s002210000504, PMID: 11104123

[ref41] Harvey D. (1989). The Condition of Postmodernity: An Enquiry into the Origins of Cultural Change. Oxford, UK: Blackwell.

[ref42] Helmholtz H. (2005). [1924] Treatise on Physiological Optics. *Vol*. 3. New York: Dover.

[ref43] Heylighen A. (2011). “Studying the unthinkable designer: designing in the absence of sight,” in Design Computing & Cognition ’10. ed. Gero J. S. (Dordrecht, the Netherlands: Springer), 23–34.

[ref44] Heylighen A. Devlieger P. Strickfaden M. (2009). “Design expertise as disability and vice versa.” in *Proceedings from the Communicating (by) Design Conference*; January 26-27, 2009; (Brussels, Belgium: Hogeschool voor Wetenschap en Kunst St.-Lucas), 227–235.

[ref45] Hoogstad J. (1990). Space-Time-Motion. Gravenhage, the Netherlands: SDU Publisher.

[ref46] Hubbard E. M. Ramachandran V. S. (2005). Neurocognitive mechanisms of synesthesia. Neuron 48, 509–520. doi: 10.1016/j.neuron.2005.10.012, PMID: 16269367

[ref47] Hurley S. L. (1998). Consciousness in Action. Cambridge, MA: Harvard University Press.

[ref48] Hurley S. L. Noë A. (2003). Neural plasticity and consciousness. Biol. Philos. 18, 131–168. doi: 10.1023/A:1023308401356

[ref49] Husserl E. (1970). The Crisis of European Sciences and Transcendental Phenomenology: An Introduction to Phenomenological Philosophy. Evanston, IL: Northwestern University Press.

[ref50] Ihde D. (2012). Experiemental Phenomenology: Multistabilities. 2nd Edn. Albany, NY: State University of New York Press.

[ref51] Ione A. Tyler C. (2003). Neurohistory and the arts: was Kandinsky a synesthete? J. Hist. Neurosci. 12, 223–226. doi: 10.1076/jhin.12.2.223.15540, PMID: 12953624

[ref52] Ionta S. Gassert R. Blanke O. (2011). Multisensory and sensorimotor foundation of bodily self-consciousness – an interdisciplinary approach. Front. Psychol. 2:383. doi: 10.3389/fpsyg.2011.00383, PMID: 22207860 PMC3245631

[ref53] Jelić A. Tieri G. De Matteis F. Babiloni F. Vecchiato G. (2016). The enactive approach to architectural experience: a neurophysiological perspective on embodiment, motivation, and affordances. Front. Psychol. 7:481. doi: 10.3389/fpsyg.2016.00481 27065937 PMC4815679

[ref54] Kandinsky V. (1982). Reminiscences. in Kandinsky: Complete Writings on Art. eds. and trans. Lindsay K. C. Vergo P. (Boston, Mass.: G. K. Hall).

[ref55] Kopec D. (2012). Environmental Psychology for Design. New York, NY: Bloomsbury Publishing.

[ref56] Korik A. Sosnik R. Siddique N. Coyle D. (2019). Decoding imagined 3D arm movement trajectories from EEG to control two virtual arms—a pilot study. Front. Neurorobot. 13:94. doi: 10.3389/fnbot.2019.00094, PMID: 31798438 PMC6868122

[ref57] Kwon J. (2017). A synesthetic approach to creative design thinking – the phenomenological perspective on multi-sensory spatial experience. *2017 IDEC Annual Conference Proceedings*, 384–390.

[ref58] Kwon J. (2018). Teaching the spatio-temporality of the body: a cross-disciplinary design exploration. *IDEC 2018 Annual Conference Proceedings*, 448–449.

[ref59] Kwon J. (2020). The corporeality of spatial experience: A kinesthetic design approach to built environments. *IDEC 2020 Annual Conference Proceedings*, 664–667.

[ref60] Kwon J. Kim J. Y. (2021). Meaning of gaze behaviors in individuals’ perception and interpretation of commercial interior environments: an experimental phenomenology approach involving eye-tracking. Front. Psychol. 12:581918. doi: 10.3389/fpsyg.2021.581918, PMID: 34484018 PMC8415749

[ref001] Lakoff G. Johnson M. (1999). Philosophy in the Flesh: The Embodied Mind and Its Challenge to Western Thought. New York: Basic Books.

[ref61] Loke L. Robertson T. (2013). Moving and making strange: an embodied approach to movement-based interaction design. ACM Trans. Comput. Hum. Interact. 20, 1–25. doi: 10.1145/2442106.2442113

[ref62] Longo M. R. Schüür F. Kammers M. P. Tsakiris M. Haggard P. (2008). What is embodiment? A psychometric approach. Cognition 107, 978–998. doi: 10.1016/j.cognition.2007.12.004, PMID: 18262508

[ref63] Lu A. Mo L. Hodges B. H. (2011). The weight of time: affordances for an integrated magnitude system. J. Exp. Psychol. Hum. Percept. Perform. 37, 1855–1866. doi: 10.1037/a0024673, PMID: 21787104

[ref64] Madden T. J. Hewett K. Roth M. S. (2000). Managing images in different cultures: a cross-national study of color meanings and preferences. J. Int. Mark. 8, 90–107. doi: 10.1509/jimk.8.4.90.19795

[ref002] Mandik P. (2005). “Action-oriented representation,” in Cognition and the Brain: The Philosophy and Neuroscience Movement. eds. Brook A. Akins K. (Cambridge University Press), 284–305.

[ref65] Massey D. (2006). Landscape as a provocation: reflections on moving mountains. J. Mater. Cult. 11, 33–48. doi: 10.1177/1359183506062991

[ref66] Meglin J. A. Eliot K. Sellers-Young B. (2018). Kinetic, kinesthetic, and modern: dance and the visual arts. Dance Chron. 41, 113–120. doi: 10.1080/01472526.2018.1470408

[ref67] Melcher D. Zampini M. (2011). “The sight and sound of music: audiovisual interactions in science and the arts,” in Art and the Senses. (Oxford, UK: Oxford University Press), 265–292.

[ref68] Melcón P. B. Romero-Naranjo J. L. Drago F. S. Romero-Naranjo F. J. (2017). Dimension analysis and architectural model of BAPNE classroom for pre-school and primary education. Procedia Soc. Behav. Sci. 237, 1284–1290. doi: 10.1016/j.sbspro.2017.02.211

[ref69] Merleau-Ponty M. (2014). *Phenomenology of Perception*. (D. A. Landes, Trans.). Abingdon and New York, NY: Routledge.

[ref70] Merter S. (2017). Synesthetic approach in the design process for enhanced creativity and multisensory experiences. Des. J. 20, S4519–S4528. doi: 10.1080/14606925.2017.1352948

[ref71] Milner A. D. Goodale M. A. (1995). The Visual Brain in Action. Oxford, United Kingdom: Oxford University Press.

[ref72] Montero B. (2012). Practice makes perfect: the effect of dance training on the aesthetic judge. Phenomenol. Cogn. Sci. 11, 59–68. doi: 10.1007/s11097-011-9236-9

[ref73] Moran D. (2000). Introduction to Phenomenology. New York, NY: Routledge.

[ref74] Mroczko-Wąsowicz A. Nikolić D. (2014). Semantic mechanisms may be responsible for developing synesthesia. Front. Hum. Neurosci. 8:509. doi: 10.3389/fnhum.2014.00509, PMID: 25191239 PMC4137691

[ref75] Nabokov (1989). Speak, Memory: An Autobiography Revisited. E-Reader Version; First Vintage International Edition. Vintage International. New York: Vintage.

[ref76] Noë A. (2010). “Vision without representation,” in Perception, Action, and Consciousness: Sensorimotor Dynamic and Two Visual Systems. eds. Gangopadhyay N. Madary M. Spicer F. (Oxford: Oxford University Press), 245–256.

[ref77] Noë A. Noë A. (2004). Action in Perception. Cambridge, Mass.: MIT Press.

[ref78] O’Regan J. K. Noë A. (2001). A sensorimotor account of vision and visual consciousness. Behav. Brain Sci. 24, 939–973. doi: 10.1023/A:1023308401356 12239892

[ref79] Pallasmaa J. (2005). The Eyes of the Skin: Architecture and the Senses. London, UK: Wiley-Academy.

[ref80] Pasqualini I. Llobera J. Blanke O. (2013). “Seeing” and “feeling” architecture: how bodily self-consciousness alters architectonic experience and affects the perception of interiors. Front. Psychol. 4:354. doi: 10.3389/fpsyg.2013.00354 23805112 PMC3691502

[ref81] Plank I. S. von Thienen J. P. A. Meinel C. (2021). “The neuroscience of empathy: research-overview and implications for human-centered design” in Design Thinking Research. Understanding Innovation. eds. Meinel C. Leifer L. (Cham: Springer)

[ref82] Ponzo S. Kirsch L. P. Fotopoulou A. Jenkinson P. M. (2018). Balancing body ownership: visual capture of proprioception and affectivity during vestibular stimulation. Neuropsychologia 117, 311–321. doi: 10.1016/j.neuropsychologia.2018.06.020, PMID: 29940194 PMC6092558

[ref83] Poulsen S. Thøgersen U. (2011). Embodied design thinking: a phenomenological perspective. CoDesign 7, 29–44. doi: 10.1080/15710882.2011.563313

[ref84] Ramachandran V. S. Hubbard E. M. (2003). The phenomenology of synaesthesia. J. Conscious. Stud. 10, 49–57.

[ref85] Riccò D. Belluscio A. Guerini S. (2003). “Design for the Synesthesia. Audio, visual and haptic correspondences experimentation.” In *Proceedings of the 1st International Meeting of Science and Technology of Design*; September 25-26, 2003; Lisbon, Portugal. 25–26.

[ref86] Schneck D. Berger D. Rowland G. (2006). The Music Effect: Music Physiology and Clinical Applications. London, UK: Jessica Kingsley Publishers.

[ref87] Schön D. A. (1983). The Reflective Practitioner: How Professionals Think in Action. New York, NY: Basic Books.

[ref88] Scruton R. (2004). Musical movement: a reply to Budd. Br. J. Aesthet. 44, 184–187. doi: 10.1093/bjaesthetics/44.2.184

[ref89] Seamon D. (1980). “Body-subject, time-space routines, and place-ballets,” in The Human Experience of Space and Place. eds. Buttimer A. Seamon D. (New York, NY: Routledge), 148–165.

[ref90] Seamon D. (1982). The phenomenological contribution to environmental psychology. J. Environ. Psychol. 2, 119–140. doi: 10.1016/S0272-4944(82)80044-3

[ref91] Seamon D. (2000). “A way of seeing people and place,” in Theoretical Perspectives in Environment-Behavior Research: Underlying Assumptions, Research Problems, and Methodologies. ed. Wapner S. (New York, NY: Kluwer Academic/Plenum Publishers), 157–178.

[ref92] Seamon D. (2015). “The phenomenological contribution to interior design education and research: place, environmental embodiment, and architectural sustenance,” in The Handbook of Interior Design. eds. Thompson J. A. J. Blossom N. H. (Chichester, UK: John Wiley & Sons), 417–431.

[ref93] Shebilske W. L. (1984). “Context effects and efferent factors in perception and cognition,” in Cognition and Motor Processes. eds. Prinz W. Sanders A. F. (Berlin: Springer-Verlag), 99–119.

[ref94] Shebilske W. L. (1987). “An ecological efference mediation theory of natural event perception,” in Perspectives on Perception and Actions. eds. Heuer H. Sanders A. F. (Hillsdale, NJ: Erlbaum), 195–213.

[ref95] Sheets-Johnstone M. (2019). Kinesthesia: an extended critical overview and a beginning phenomenology of learning. Cont. Philos. Rev. 52, 143–169. doi: 10.1007/s11007-018-09460-7

[ref96] Sherrington C. (1906). The Integrative Action of the Nervous System. New York, NY: C. Scribner’s Sons.

[ref97] Shima X. Sato R. (2017). A novel haptic device design based on somatosensory superimposed stimuli. Adv. Robot. 31, 135–142. doi: 10.1080/01691864.2016.1266093

[ref98] Simonsen K. (2005). Bodies, sensations, space and time: the contribution from Henri Lefebvre. Geogr. Ann. Ser. B 87, 1–14. doi: 10.1111/j.0435-3684.2005.00174.x

[ref99] Sun H.-J. Campos J. L. Young M. Chan G. S. W. Ellard C. G. (2004). The contributions of static visual cues, non-visual cues, and optic flow in distance estimation. Perception 33, 49–65. doi: 10.1068/p5145, PMID: 15035328

[ref100] Svanæs D. (2013). Interaction design for and with the lived body: Some implications of Merleau-Ponty's phenomenology. ACM Trans. Comput. Hum. Interact. 20, 1–30. doi: 10.1145/2442106.2442114

[ref101] Torrents C. Castañer M. Jofre T. Morey G. Reverter F. (2013). Kinematic parameters that influence the aesthetic perception of beauty in contemporary dance. Perception 42, 447–458. doi: 10.1068/p7117, PMID: 23866557

[ref102] Tversky B. (2005). “Functional Significance of Visuospatial Representations,” in The Cambridge Handbook of Visuospatial Thinking. eds. Shah P. Miyake A. (Cambridge, United Kingdom: Cambridge University Press), 1–34.

[ref103] Tversky B. (2019). Mind in Motion: How Action Shapes Thought. UK: Hachette.

[ref104] Valenzuela-Moguillansky C. Reyes-Reyes A. Gaete M. I. (2017). Exteroceptive and interoceptive body-self awareness in fibromyalgia patients. Front. Hum. Neurosci. 11:117. doi: 10.3389/fnhum.2017.00117, PMID: 28348526 PMC5346579

[ref105] Van Campen C. (2008). The Hidden Sense: Synesthesia in Art and Science. Cambridge, MA: The MIT Press.

[ref106] Van Campen C. (2011). “Visual music and musical paintings: the quest for synaesthesia in the arts,” in Art and the Senses. eds. Melcher D. Bacci F. (Oxford, UK: Oxford University Press), 495–512.

[ref107] van Leeuwen T. M. Dingemanse M. Todil B. Agameya A. Majid A. (2016). Nonrandom associations of graphemes with colors in Arabic. Multisens. Res. 29, 223–252. doi: 10.1163/22134808-00002511, PMID: 27311298

[ref108] Van Manen M. (1997). Researching Lived Experience: Human Science for an Action Sensitive Pedagogy. 2nd Edn. London, UK: The Althouse Press.

[ref109] Van Rompay T. J. L. Ludden G. D. S. (2015). Types of embodiment in design: the embodied foundations of meaning and affect in product design. Int. J. Des. 9, 1–11.

[ref110] Ward J. Li R. Salih S. Sagiv N. (2007). Varieties of grapheme-colour synaesthesia: a new theory of phenomenological and behavioural differences. Conscious. Cogn. 16, 913–931. doi: 10.1016/j.concog.2006.09.012, PMID: 17126034

[ref111] Wastiels L. W. Schifferstein H. J. Wouters I. Heylighen A. (2013). Touching materials visually: about the dominance of vision in building material assessment. Int. J. Des. 7, 31–41.

[ref112] Wilde D. Schiphorst T. Klooster S. (2011). Move to design/design to move: a conversation about designing for the body. Interactions 18, 22–27. doi: 10.1145/1978822.1978828

[ref113] Wittkower R. (1971). Architectural Principles in the Age of Humanism. London, UK: A. Tiranti.

[ref114] Yoon J. (2008). Searching for an image conveying connotative meanings: an exploratory cross-cultural study. Libr. Inf. Sci. Res. 30, 312–318. doi: 10.1016/j.lisr.2008.04.004

[ref115] Zumthor P. (2010). Thinking Architecture. 3rd Edn. Basel, Switzerland: Birkhäuser.

